# Comparing Two Web-Based Smoking Cessation Programs: Randomized Controlled Trial

**DOI:** 10.2196/jmir.993

**Published:** 2008-11-18

**Authors:** H Garth McKay, Brian G Danaher, John R Seeley, Edward Lichtenstein, Jeff M Gau

**Affiliations:** ^1^Oregon Research InstituteEugeneORUSA

**Keywords:** Tobacco, smoking cessation, Web-assisted tobacco intervention, Internet, intervention, adherence, engagement, physical activity

## Abstract

**Background:**

Smoking cessation remains a significant public health problem. Innovative interventions that use the Internet have begun to emerge that offer great promise in reaching large numbers of participants and encouraging widespread behavior change. To date, the relatively few controlled trials of Web-based smoking cessation programs have been limited by short follow-up intervals.

**Objective:**

We describe the 6-month follow-up results of a randomized controlled trial in which participants recruited online were randomly assigned to either a Web-based smoking cessation program (Quit Smoking Network; QSN) or a Web-based exercise enhancement program (Active Lives) adapted somewhat to encourage smoking cessation.

**Methods:**

The study was a two-arm randomized controlled trial that compared two Web-based smoking cessation programs: (1) the QSN intervention condition presented cognitive-behavioral strategies, and (2) the Active Lives control condition provided participants with guidance in developing a physical activity program to assist them with quitting. The QSN condition provided smoking cessation information and behavior change strategies while the Active Lives condition provided participants with physical activity recommendations and goal setting. The QSN condition was designed to be more engaging (eg, it included multimedia components) and to present much greater content than is typically found in smoking cessation programs.

**Results:**

Contrary to our hypotheses, no between-condition differences in smoking abstinence were found at 3- and 6-month follow-up assessments. While participants in the QSN intervention condition spent more time than controls visiting the online program, the median number of 1.0 visit in each condition and the substantial attrition (60.8% at the 6-month follow-up) indicate that participants were not as engaged as we had expected.

**Conclusions:**

Contrary to our hypothesis, our test of two Web-based smoking cessation conditions, an intervention and an attention placebo control, failed to show differences at 3- and 6-month assessments. We explored possible reasons for this finding, including limited engagement of participants and simplifying program content and architecture. Future research needs to address methods to improve participant engagement in online smoking cessation programs. Possible approaches in this regard can include new informed consent procedures that better explain the roles and responsibilities of being a research participant, new program designs that add more vitality (changing content from visit to visit), and new types of reminders pushed out to participants to encourage return visits. Simplifying program content through a combination of enhanced tailoring and information architecture also merits further research attention.

## Introduction

The importance of smoking on public health is undeniable: “Cigarette smoking remains the leading cause of preventable mortality in the United States, resulting in nearly 16 million deaths since the first Surgeon General’s report on smoking and health in 1964” [1]. In addition to the profound deleterious impact on smokers’ health, “secondhand smoke causes premature death and disease in children and in adults who do not smoke” [2,3]. In response to the public health risks associated with smoking, numerous research projects have examined tobacco cessation approaches that are based in clinical settings as well as public health methods that permit wider dissemination [4-6].

One nascent area of development for public health smoking cessation programs involves the use of Internet-based interventions. To date, only a handful of published studies have described the efficacy/effectiveness of Web-based tobacco cessation programs. Several reports have described promising results of trials with participants from commercial or tobacco control agency Web-based programs for smokers [7-10]. Other published studies have described initial feasibility studies of Web-based tobacco cessation programs [11,12]. A few randomized controlled trials (RCTs) have been conducted [9,13-19], and their results have been generally encouraging [20]. However, interpretation of the randomized trials is complicated by short follow-up intervals, for example, 6 weeks in Strecher et al [16], 2.5 months in Etter [8], and 3 months in Lenert et al [15], Pike et al [9], and Swartz et al [18]. Additional trials with longer term follow-up assessments examining the use of Web-based interventions for smoking control are clearly warranted.

The current paper describes results of the Smokers’ Health Improvement Program (SHIP) RCT that examined the 3- and 6-month outcomes for two Web-based programs: (1) an intervention condition (Quit Smoking Network; QSN) that provided users with extensive information and behavioral strategies drawn from clinic-based and self-help smoking cessation programs, or (2) a Web-based control condition that focused on increasing physical activity (Active Lives). Currently, because grant funding has ended, neither the QSN nor Active Lives website is currently available for review. However, screenshots of the programs are included in the Multimedia Appendix.

In addition to primary outcome measures of tobacco abstinence, we sought to examine the impact of condition on secondary outcomes, including participant exposure to program content, physical activity, and pharmacotherapy use. We also sought to test the putative predictors of outcome. Finally, we assessed the extent to which participants found their assigned program easy to use.

## Methods

### Study Design

The SHIP study was a two-arm RCT that compared two fully automated, Web-based smoking cessation programs: (1) the QSN intervention condition that presented cognitive-behavioral strategies, and (2) the Active Lives control condition in which participants received guidance in developing a personal fitness program to help them quit smoking. The QSN condition provided participants with smoking cessation information and behavior change recommendations while the Active Lives condition provided participants with physical activity recommendations, monitoring, and goal setting. The QSN condition was designed to be more engaging (it included video testimonials, for example), and it presented much greater content that is more typically found in smoking cessation programs. The Active Lives program provided content more explicitly aimed at increasing physical activity as a smoking cessation approach, and, although there is some evidence that physical activity adjuncts to smoking cessation programs may be helpful (eg, [21]), the Active Lives condition was intended to be a credible attention placebo control condition. Follow-up assessments occurred at 3 and 6 months following program enrollment. The trial was not registered, because enrollment started in spring 2005, before trial registration became mandatory.

### Enrollment and Participants

**Figure 1:**
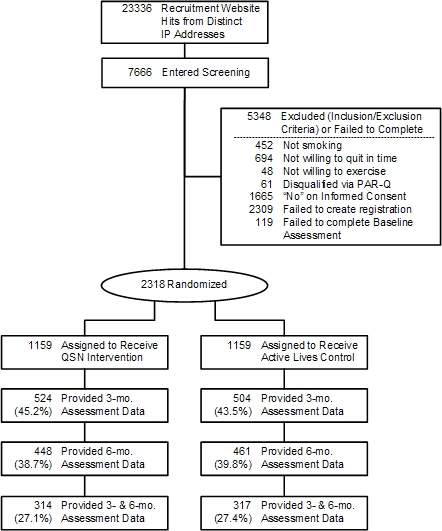
CONSORT diagram depicting flow of study participants

We first sought to recruit participants through large worksites in order to reach our recruitment goals and to minimize attrition. This strategy proved unsuccessful. In consultation with an Internet marketing firm, we designed and executed a purely Internet-based recruitment campaign. The campaign involved ad placement on Google and Yahoo search engines (keywords “quit smoking” and “stop smoking”) and links to their relevant affiliated sites. Clicking our ads enabled users to (1) visit our recruitment site (study description, inclusion/exclusion criteria), (2) submit answers to screening items, (3) provide their informed consent, and (4) complete the baseline assessment. This Internet marketing campaign was remarkably successful: we recruited 2318 participants in only 10 weeks at a cost of approximately US $13 per recruit. A total of 69.8% (1169/2318) came from Google, 19.9% (461/2318) from Yahoo, and the remaining 10.3% (238/2318) were recruited from word of mouth or from unknown other sources. The flow of participants across various study milestones is depicted using a CONSORT diagram in Figure 1. Note that 44.3% (1028/2318) of participants completed the 3-month follow-up assessment, 39.2% (909/2318) completed the 6-month assessment, and 631 (27.2%) of the randomized sample completed both the 3- and the 6-month assessments.

Study inclusion criteria were as follows: (1) at least 18 years of age, (2) current smoker interested in quitting within the next 30 days, (3) willingness to engage in moderate physical activity, (4) access to the Internet, (5) agreement with the informed consent statement as approved by the Institutional Review Board of Oregon Research Institute, and (6) completion of both program registration and the baseline assessment. Exclusion criteria included any positive answers on the 8-item Physical Activity Readiness Questionnaire (PAR-Q), designed to identify individuals for whom physical activity might be inappropriate or who should receive medical advice concerning the type of activity most suitable for them. Example exclusion items included “Has your doctor ever said you have heart trouble?” and “Do you often feel faint or have spells of severe dizziness?” [22].

Screening and baseline assessments were used to measure the characteristics of participants, including age, gender, race/ethnicity, current smoking, rurality [23], and education. Study participants were predominantly white, urban, 30- to 50-year-old married women who had at least some college education and smoked 1-2 packs of cigarettes per day at baseline (see participant characteristics in Table 1). No statistically significant differences were found between conditions on any participant characteristics.

**Table 1:** Distribution of baseline participant characteristics

Characteristic	QSN No. (%)	Active Lives No. (%)	Total No. (%)
**Age (years)**			
< 30	266 (23.0)	253 (21.8)	519 (22.4)
30-39	256 (22.1)	286 (24.7)	542 (23.4)
40-49	360 (31.1)	327 (28.2)	687 (29.6)
≥ 50	277 (23.9)	293 (25.3)	570 (24.6)
**Female**	805 (69.5)	829 (71.5)	1634 (70.5)
**Married**	697 (60.1)	730 (63.0)	1427 (61.6)
**Race/Ethnicity**			
White	990 (86.8)	982 (86.4)	1972 (86.6)
Black	78 (6.8)	76 (6.7)	154 (6.8)
Other	72 (6.3)	79 (6.9)	151 (6.6)
**Education**			
No high school degree	79 (6.8)	80 (6.9)	159 (6.9)
High school graduate	302 (26.1)	276 (23.8)	578 (24.9)
Some college	453 (39.1)	490 (42.3)	943 (40.7)
College graduate	325 (28.0)	313 (27.0)	638 (27.5)
**Rural vs urban**	226 (19.9)	220 (19.5)	446 (19.7)
**Number of cigarettes smoked/day**			
≤ 10	194 (16.8)	199 (17.2)	100 (17.0)
11-20	497 (42.9)	458 (39.5)	955 (41.3)
21-40	423 (36.5)	442 (38.2)	865 (37.4)
≥ 41	44 (3.8)	57 (4.9)	101 (4.4)

### Description of the Web-Based Programs

#### QSN Intervention Condition

The QSN condition incorporated a hybrid information architecture [24] in which first-time users were directed through a series of tailored Web pages (tunnel design) in order to introduce them to the key concepts and strategies of a behavioral program for quitting smoking. Once they emerged from the tunnel, users were able to choose their own path to access a broad array (using a matrix design) of additional content on quitting and maintaining nonsmoking.

Components of the smoking cessation intervention used in the study are based on Social Cognitive Theory [25,26] as it has been applied to tobacco abstinence [27,28]. These components are designed to help encourage tobacco abstinence via the use of strategies that address each participant’s behavior, cognition, and environment [29,30]. This approach also builds on behavioral self-management [31,32], in which the intervention is viewed as providing structure, skills, and a supporting scaffold that encourage the participant to become an active problem solver in the iterative process of trying out and then refining the use of a series of strategies as a part of a personalized plan for quitting tobacco.

Key content modules focused on getting ready, developing a personal quitting plan, setting a personal quit date, using pharmacotherapy products including nicotine replacement therapy, avoiding and altering trigger situations, using substitutes, managing thoughts, using strategies to manage mood, and obtaining support from a peer-to-peer Web forum as well as a professionally moderated “Ask an Expert” forum. The program also offered an extensive library of additional content. Because users were required to log in to the website using their unique username and password, we were able to tailor portions of the program content to each participant’s smoking/nonsmoking status (checked at the start of each session) and to display online prompts recommending the review of program content that a participant had not yet explored.

#### Active Lives Control Condition

Participants assigned to the Active Lives control condition accessed a Web-based program designed to encourage them to engage in a personalized fitness program that would help them quit smoking. The Active Lives program was based primarily on Bandura’s Social Cognitive Theory [25,26], research on interventions to promote physical activity [33,34], and our earlier research on an online diabetes self-management intervention [35]. The program guided each participant through a multi-step plan that included a motivational component (exploration of the benefits of physical activity and a clarification of personal goals and barriers), a behavioral action plan with extensive tracking features (eg, weekly activity schedules personalized to each participant’s schedule and types of activities), additional online resources (articles and tips sheets), and access to a Web forum for peer support.

### Method of Assessment (Online and Phone)

We attempted to collect all participant assessments via the Internet. Participants were sent an email reminder 3 days prior to an assessment and on the due date of that assessment. Participants who failed to complete their online assessment within 1 week were sent an additional email reminder. If participants had not completed their online assessment within a 2-week period, then a project research assistant initiated a process to complete assessments by phone.

### Primary Outcome Measures: Smoking Cessation

Following the recommendation of the Society of Research on Nicotine and Tobacco [36], we assessed point prevalence smoking/nonsmoking status by asking “Have you smoked any cigarettes in the last week, even a puff?” Each participant’s smoking abstinence was measured at 3 and 6 months post-enrollment. In addition, we examined repeated point prevalence nonsmoking at both the 3- and 6-month assessments. Because our intervention was accomplished entirely online without any personal contact between participant and researchers, we concluded that it was impractical to obtain biochemical validation of self-reported abstinence—a decision consistent with both the recommendations of Glasgow et al [37] for low-intensity intervention trials as well as with many of the published trials of Web-based smoking cessation programs (eg, [7,13,18]).

### Putative Predictors

In addition to baseline demographic and smoking data (see Table 1), the set of putative predictors we planned to examine included self-efficacy, dependence, support for quitting, smoking among friends and family members, depression, and prior quit attempts. Tests of putative predictors help to establish the veracity of the dataset because they broaden the knowledge base and enable comparisons with other similar findings in the literature. Reporting predictors could help inform future intervention design in showing characteristics of those participants for whom the programs seemed most efficacious. Examples of studies that have examined predictors of outcome using participants who had participated in a tobacco cessation RCT include those performed by Oregon Research Institute researchers [19,38,39] and other research teams (eg, [8,15,40-43]).

#### Self-Efficacy

Confidence in accomplishing various facets of quitting smoking was assessed at baseline using a 5-point Likert scale (1 = not at all confident to 5 = very confident). Items included the following: “If you decided to quit smoking, how confident are you that you could quit?”, “If you decided to quit smoking, how confident are you that you will not be smoking a year from now?”, “How confident are you that you can resist smoking when you are feeling bored or restless?”, “How confident are you that you can resist smoking when you are angry, frustrated, or tense?”, “How confident are you that you can resist smoking when you drink alcohol?”, and “How confident are you that you can resist smoking when you are around others who are also using it?

#### Dependence

Nicotine dependence was measured at baseline using an item excerpted from a scale developed by Piper et al [44]: “How strong are your urges when you first wake up in the morning?” This was assessed with a 7-point Likert scale (1 = not strong at all to 7 = extremely strong). In addition, participants were asked at baseline to answer the following question using one of eight answer options (1-5, 6-10, 11-15, 16-20, 21-25, 26-30, 31-40, 40 or more): “On average, how many cigarettes do you smoke each day?”

#### Support for Quitting

Participants were asked at baseline to rate the expected support for quitting: “If you decided to quit smoking, how supportive would the person you’re closest to be of your efforts to stop smoking?” (1 = not at all supportive to 7 = very supportive).

#### Smokers Among Friends and Family

Participants were asked to answer two items recommended by Piper et al [44] using a 7-point Likert scale (1 = not true of me at all to 7 = extremely true of me): “A lot of my friends or family smoke” and “Most of my friends and acquaintances smoke.”

#### Depression

The baseline assessment asked participants to answer two dichotomous (yes/no) items that measured depression: “In the past year, have you had 2 weeks or more during which you felt sad, blue, or depressed; or when you lost all interest or pleasure in things that you usually cared about?” and “Have you had 2 years or more in your life when you felt depressed or sad most days, even if you felt okay sometimes?”

#### Quit Attempts

Participants were asked to answer the question “In the past year, how many times have you made a serious attempt to quit?” using five answer options: 0, 1, 2, 3, or ≥ 4 attempts.

### Secondary Outcome Measures

#### Participant Exposure

The extent to which participants accessed their assigned Web-based program was measured unobtrusively using a combination of database tracking and Web server log analysis [45] to determine both number of visits (sessions) and duration of visits. We also created a composite measure of exposure (mean of standard scores for the number of visits and total time spent across all visits). We examined the pattern of declining participant visits to the Web-based programs following enrollment [45] by calculating each participant’s final visit date to view program content (visits associated with completing online assessments were not included). If a participant viewed program content only one time and it occurred on his/her enrollment date, then he/she would be assigned 0 days (last visit occurred zero days since the day of enrollment).

#### Physical Activity

Two items from the Behavioral Risk Factor Surveillance System (BRFSS) [46] were used to measure whether participants engaged in vigorous or moderate levels of continuous activity: “In a usual week, do you do vigorous activities for at least 10 minutes at a time, such as running, aerobics, heavy yard work, or anything else that causes large increases in breathing or heart rate?” and “In a usual week, do you do moderate activities for at least 10 minutes at a time, such as brisk walking, bicycling, vacuuming, gardening, or anything else that causes some increase in breathing to heart rate?” Endorsement of these items was followed by the following question: “How many days per week do you do vigorous [moderate] activities?”

#### Pharmacotherapy Use

Participants in both conditions were asked the following question on the 3- and 6-month assessments: “Which of the following products or methods have you tried in the last 3 months?” Answer options included nicotine gum, nicotine patches, nicotine lozenges, nicotine spray, nicotine inhaler, other nicotine replacement product, and Zyban (bupropion). Two composite scores were derived for each participant: the sum of pharmacotherapy products used (from 0 to 6) and a yes/no score for any pharmacotherapy use.

#### Program Usability

At each of the 3- and 6-month assessments, participants were asked to answer “How easy was it to use the QSN/Active Lives program?” using a 5-point Likert scale (1 = not at all; 3 = somewhat; 5 = very).

### Statistical Analyses

#### Logistic Regression and Survival Analysis

Putative predictors were tested using two complementary steps. First, we used binary logistic regression models that incorporated treatment condition as well as the interaction of the condition with each variable in order to identify any differential effects of the intervention on the prediction of smoking abstinence [47]. Second, significant predictors were then tested in a multivariate binary logistic regression using backwards elimination. Following the approach we used in another Web-based tobacco cessation program [45], we used Kaplan-Meier survival analyses [48,49] to examine the pattern of last visits to the Web-based program using the number of days post-enrollment as our unit of time. All analyses were conducted using SPSS statistical software, version 15 (SPSS Inc, Chicago, IL, USA).

#### Complete Case vs Intent-to-Treat Analyses

Given that only 39.2% (909/2318) of participants responded to the 6-month follow-up assessment, we decided not to use complex imputation methods [50]. Instead, we used two complementary approaches: (1) complete case analysis limited to data obtained from participants who responded to a follow-up assessment, and (2) an intent-to-treat analysis that used the original sample as the denominator and a simple imputation method in which all participants who did not complete an assessment were considered to be still smoking (missing = smoking).

## Results

### Assessment Completion / Participant Attrition

Of the 1028 participants who completed the 3-month assessment, 315 (30.6%) did so online and 713 (69.4%) were contacted by phone. Of the 909 participants who completed the 6-month assessment, 161 (17.7%) completed it online and 748 (82.3%) completed a phone assessment. The two conditions did not differ in terms of the proportion of online assessments at 3 months, but QSN had more online 6-month assessments than the Active Lives condition: 21.4% (96/448) vs 14.1% (65/461); *χ* ^2^_1_ = 8.34, N = 909, *P* = .004.

We used multivariate logistic regression on complete cases to examine possible baseline predictors using two dependent variables: (1) completion of the 6-month assessment and (2) completion of both the 3- and 6-month assessments. Positive predictors of completing the 6-month assessment included age (adjusted odds ratio [OR] = 1.33, 95% confidence interval [CI] = 1.23-1.44, *P* < .001), education level (OR = 1.18, CI = 1.07-1.29, *P* = .001), and confidence in being able to quit for one year (OR = 1.08, CI = 1.00-1.15, *P* = .04). Positive predictors of completing both the 3- and 6-month assessments included age (OR = 1.40, CI = 1.30-1.51, *P* < .001), marital status (OR = 1.13, CI = 1.05-1.48, *P* = .01), education level (OR = 1.13, CI = 1.03-1.24, *P* = .01), and confidence in being able to quit for one year (OR = 1.10, CI = 1.03-1.18, *P* = .008). None of the condition by predictor tests reached statistical significance.

### Primary Outcome: Tobacco Cessation

Self-reported smoking abstinence was examined by condition using complete case and intent-to-treat analyses at 3 months, 6 months, and also for both the 3- and 6-month follow-up assessments (see Table 2). Results for the QSN and Active Lives conditions were remarkably similar.

Binary logistic regression tests (complete case) failed to uncover any significant differences in smoking abstinence between the QSN and Active Lives conditions when we considered assessments at 6 months or when we considered nonsmoking at both 3 and 6 months (see Table 3). Intent-to-treat (missing = smoking) analyses showed similar nonsignificant between-group differences.

**Table 2:** Smoking abstinence by condition at follow-up assessments

	3 Months No. (%)	6 Months No. (%)	3 and 6 Months No. (%)
**Complete Case**			
QSN	103/524 (19.7)	112/448 (25.0)	45/314 (14.3)
Active Lives	99/504 (19.6)	120/461 (26.0)	44/317 (13.9)
**Intent-to-Treat**			
QSN	103/1159 (8.9)	112/1159 (9.7)	45/1159 (3.9)
Active Lives	99/1159 (8.5)	120/1159 (10.4)	44/1159 (3.8)

**Table 3:** Logistic regression results of smoking abstinence by condition at follow-up assessments

	*β*	OR	95% CI		*P*
			Lower	Upper	
**3 Months**					
Complete case	−.001	1.00	.73	1.36	.996
Intent-to-treat	−.043	.96	.72	1.28	.768
**6 Months**					
Complete case	.054	1.06	.78	1.42	.722
Intent-to-treat	.077	1.08	.82	1.42	.580
**Both 3 and 6 Months**					
Complete case	−.037	.96	.62	1.51	.871
Intent-to-treat	−.023	.98	.64	1.49	.914

### Predictors of Outcome

None of the interactions between putative predictors and treatment condition were significant. Results of multivariate binary logistic tests performed on smoking abstinence for all participants (collapsed across condition) at 3 months, 6 months, and both 3 and 6 months are presented in Table 4. Education emerged as a significant positive predictor of smoking abstinence in all three cases. Baseline smoking rate was negatively related to smoking abstinence on each of the single follow-up assessments. Expected support for quitting had a positive relation with smoking abstinence at the 3-month assessment and the combined 3- and 6-month assessment.

**Table 4:** Predictors of smoking abstinence by follow-up assessment

	*β*	OR	95% CI		*P*
			Lower	Upper	
**3 Months**					
Baseline cigs/day	−.125	.88	.80	.97	.010
Education	.408	1.50	1.24	1.83	.000
Expected support	.211	1.24	1.10	1.39	.001
Marital status	.415	1.52	1.07	2.14	.019
**6 Months**					
Baseline cigs/day	−.200	.82	.75	.90	.000
Education	.267	1.31	1.09	1.57	.004
**Both 3 and 6 Months**					
Education	.340	1.41	1.06	1.86	.018
Expected support	.205	1.23	1.02	1.48	.032
Serious quit attempts	−.121	.81	.67	.98	.030

### Secondary Outcomes

#### Participant Exposure

**Figure 2:**
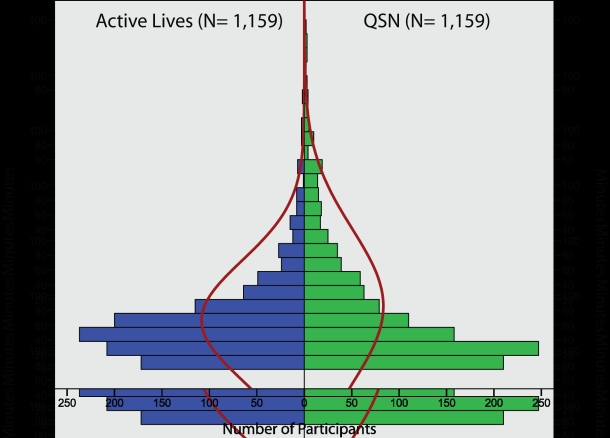
Total time (minutes) of Web program access by condition (red line indicates normal distribution)

The frequency and duration of each participant’s visits to the Web-based program (not including visits to complete online assessment) were measured unobtrusively in Web server logs and using database tracking methods (see Table 5 and Figure 2). Compared to the Active Lives condition, participants in the QSN condition averaged more visits (2.14 visits, SD = 3.66 vs 1.74 visits, SD = 2.43; unequal variance *t*_2012.03_ = −3.11, *P* = .002). Analysis of data on duration of program usage by condition (Figure 2) revealed that the QSN condition was notably less kurtotic and skewed than the Active Lives condition (kurtosis = 21.79 vs 181.70; skewness = 3.53 vs 10.38). Analysis of these data revealed that participants in the QSN condition spent significantly more time visiting the program (18.04 minutes, SD = 22.18 vs 14.02 minutes, SD = 17.09; unequal variance *t*_2174.56_ = −4.02, *P* < .001).

**Table 5:** Exposure by condition (IQR: Intraquartile Range)

	Number of Visits				Duration of Visits (minutes)			
	Mean	SD	Median	IQR	Mean	SD	Median	IQR
QSN	2.14^a^	3.66	1.00	1 (1-2)	18.04^b^	22.18	10.00	19 (5-24)
Active Lives	1.74	2.43	1.00	1 (1-2)	14.02	17.09	11.00	11 (6-17)

^a^Between-condition comparison: *P* = .001.

^b^Between-condition comparison: *P* < .001.

**Figure 3:**
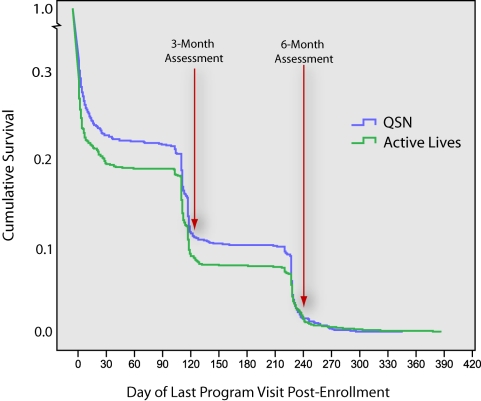
Survival analysis of program engagement over time by condition

We examined the pattern of participant exposure over time by condition. For purposes of this analysis, we defined exposure as the number of days elapsed between each participant’s date of randomization/enrollment and the date of his/her last website visit to view program content. We applied the Kaplan-Meier survival analysis to these data, which allowed us to examine the timing of the last visit by condition (see Figure 3). By definition, all participants had a last visit since all participants stopped visiting at some point following enrollment. Note that Figure 3 shows a steep downward slope in last program visits soon after program enrollment, indicating that most participants stopped visiting the program soon after they started. There were notable drops in subsequent participation at times that corresponded with the follow-up assessments. While QSN had somewhat longer estimated survival time (mean = 36.71 days, SE = 2.18) than the Active Lives condition (mean = 30.86, SE = 2.02), the Kaplan-Meier survival tests revealed that the overall trajectory of these post-enrollment program visit curves did not significantly differ by condition: Breslow (2.30, *P* = .13) and Log Rank Mantel-Cox (1.97, *P* = .16).

Binary logistic regression analyses failed to reveal any significant dose-response effects using the composite exposure score (incorporating both number of visits and total usage duration) and smoking abstinence at both 3 and 6 months within and combined for conditions (complete case analyses).

#### Physical Activity

At the 6-month assessment, remarkably similar proportions of participants in the QSN and Active Lives conditions reported that they engaged in vigorous physical activity—40.2% (173/430) vs 38.0% (163/429), respectively—and moderate physical activity—76.7% (332/433) vs 79.4% (344/433), respectively. No significant group differences were found in the reported number of days the participant had engaged in at least 10 minutes of moderate or vigorous physical activity (group *t* values ranged from .13-1.33). Number of days of vigorous activity tended to increase slightly across the baseline, 3-month, and 6-month assessments for both the QSN (mean = 3.42, SD = 1.52; mean = 3.74, SD = 1.51; mean = 3.69, SD = 1.46; respectively) and Active Lives conditions (mean = 3.47, SD = 1.62; mean = 3.51, SD = 1.65; mean = 3.85, SD = 1.49; respectively). The days of moderate activity were slightly higher for each group and also increased slightly across the baseline, 3-month, and 6-month assessments for both the QSN (mean = 3.93, SD = 1.75; mean = 4.40, SD = 1.91; mean = 4.44, SD = 1.83; respectively) and Active Lives conditions (mean = 4.01, SD = 1.76; mean = 4.42, SD = 1.80; mean = 4.33, SD = 1.88; respectively).

#### Pharmacotherapy Use

Between-group differences in terms of the number of pharmacotherapy products participants reported using were not significant at the 3-month assessment (mean = .68, SD = .86 vs mean = .60, SD = .83; unequal variance *t*_1026.97_ = −1.54, *P* = .12), but participants in the QSN condition reported using significantly more pharmacotherapy products than those in the Active Lives condition at the 6-month assessment (mean = .06, SD = .82 vs mean = .55, SD =.71; unequal variance *t*_880.06_ = −1.76, *P* = .04, 1-tailed).

We also examined pharmacotherapy use using a composite variable that measured any use (yes/no). At the 3-month assessment, significantly more QSN participants reported using any pharmacotherapy than did the Active Lives participants: 54.2% (262/524) vs 45.8% (221/504); *χ* ^2^_1_ = 3.90, N = 1028, *P* = .048. However, no group-wise differences emerged on this dimension at the 6-month assessment, with pharmacotherapy use reported by 47.3% (212/448) of QSN participants compared with 43.8% (202/461) of Active Lives participants.

#### Program Usability

 We analyzed ratings of program ease of use obtained at the 3-month assessment from 67.0% (351/524) of QSN participants and 72.6% (366/504) of Active Lives participants. Results favored QSN over the Active Lives control condition (mean = 3.85, SD = 1.28 vs mean = 3.65, SD = 1.36; with a rating of 3 = somewhat; unequal variance *t*_714.79_ =−2.05, *P* = .04).

Usability ratings obtained at the 6-month assessment from 79.0% (354/448) of QSN participants and 60.1% (277/461) of Active Lives participants showed a similar relative advantage for the QSN condition: mean = 4.10, SD = 1.21 vs mean = 3.70, SD = 1.35; unequal variance *t*_629_ = −3.91, *P* < .001.

It is important to note that participants were also asked to rate how helpful they found specific program areas (eg, library of materials and the support group area), but we chose not to report these data because very few individuals provided data on these items and a number of those participants who did provide such ratings did not, in fact, visit the program area based upon our unobtrusive tracking of their use of their assigned website.

## Discussion

The outcome results did not support our hypothesis that the QSN online smoking cessation intervention would be more effective than a credible control condition. The unremarkable impact of the QSN condition relative to the Active Lives condition is particularly surprising given that the Active Lives control condition presented very few strategies for quitting smoking since it was largely focused on helping participants improve their personal level of physical activity. The absolute level of nonsmoking at 6 months—less than 4% abstinence using intent-to-treat analysis—is less than results for other Web-based smoking cessation programs reported by Muñoz et al (5.6% to 26.0% abstinence at 6 months) [13]. However, with respect to the nonsignificant finding between conditions, the engagement of physical activity among the Active Lives participants was noteworthy. These data reflect their relatively high level of adherence to the recommended behavior change goal. Almost 80% of participants reported that they were engaged in moderate physical activity at the 6-month follow-up assessment. This implies that our control group was actively following the recommendations suggesting the importance of engaging in physical activity in aiding quit attempts and might explain, in part, the relative lack of difference in findings between the groups on quit success.

There are noteworthy strengths and limitations to consider when interpreting these findings. Strengths include the large sample of 2318 participants, the fact that the Web-based programs were fully automated to assure high fidelity of content delivery, and that the study used a RCT methodology.

One important limitation involved participant attrition (failure to complete follow-up assessments). At the 3-month follow-up, we experienced a 55.6% (1289/2318) attrition rate, which is larger than the 44% attrition rate reported by Swartz et al [18] but comparable to the levels reported for other Web-based smoking cessation programs: 57.2% by Stoddard et al [11], 62.5% by Strecher et al [16], 70.7% by Cobb et al [7], and 64.6% by Etter [8]. At the 6-month follow-up, we experienced a 60.8% (1409/2318) attrition rate, which is roughly comparable to the attrition at 6 months reported by Muñoz et al [13] for four studies of English- and Spanish-language Web-based smoking cessation programs: 73.9% (2051/2774), 69.9% (491/702), 65.7% (184/280), and 35.4% (102/288).

Although mean program exposure measures (especially duration) favored the QSN program when compared with the Active Lives control condition, the extent of these observed differences was not as large as had been expected. Nor did we find a dose-response relationship between program exposure and smoking abstinence at follow-up. Using median data, participants in the current study used the program for a single visit that lasted about 10 minutes.

Differences in study design among published studies of Web-based smoking cessation interventions and the nascent stage of the science make it difficult to generalize our program exposure results to the available body of research in this area. For example, some researchers have not reported exposure data [13,15,18], while others [8] reported number of pages viewed and average visit duration but not a precise calculation of exposure. The pattern of program visits (mean = 2.14, median = 1.0) observed for both conditions in this study is lower than the findings reported by Swartz et al [18] and Lenert et al [14] but roughly consistent with results of other online cessation programs reported by Muñoz et al [13]. In their comparison of five different Web-based smoking cessation interventions, Pike et al [9] noted that the two websites with the highest “utilization rates” had 9.7 and 6.0 visits per participant, while the visit rate on the remaining websites ranged from 1.8 to 2.2 visits.

It is important to note that Japuntich et al [51] reported much higher levels of exposure to an adjunctive Web-based intervention by participants who had multiple in-person contacts with research project staff (including a physical exam, several counseling visits, and five in-person follow-up assessments). It is reasonable to assume that there are likely to be significant differences between adjunctive use of Web-based interventions compared to fully automatic Web-based interventions like ours that do not involve any in-person contact and that deliver the intervention only by a rules-driven algorithm.

Nonetheless, there remains the opportunity to improve participant engagement in online smoking cessation programs. For example, future research should consider testing whether engagement might be facilitated by changing program content from visit to visit (enhancing vitality), by using more effective tailoring to improve the relevance of program content, and/or by using innovative reminders (eg, some combination of email, regular mail, text messages, e-cards) that encourage multiple program visits.

Analyses of pharmacotherapy usage were supportive of the fact that the QSN condition compared to the control condition encouraged significantly greater use of this treatment approach (more than 60% vs approximately 47%), although it is important to acknowledge that almost half of participants in the control condition used pharmacotherapy without being explicitly told to do so. Similarly, Swartz et al [18] reported that a majority of wait-list control participants indicated that they used pharmacotherapy products.

One interesting possibility is that our use of a physical activity control condition may have inadvertently jeopardized, to some extent, the generalizability of our results. Specifically, because of concerns about health among participants in the Active Lives condition, we excluded 61 individuals (prior to randomization) who had positive answers on the PAR-Q assessment, and an additional 48 individuals declined to participate because they did not want to increase their level of exercise activity. These 109 individuals would not have been excluded from a typical smoking cessation RCT. However, it remains for further research to determine whether these exclusion criteria may have had the effect of excluding individuals who otherwise would have been successful quitters using the treatment condition.

We considered possible control/comparison conditions for the present study. A no-treatment or waiting list control group is often recommended in order to reduce the likelihood of a type II (false negative) error when an intervention is expected to produce small effects. But we concluded that offering participants no treatment or delayed treatment would have been unhelpful because waiting list controls provide only limited information [52]. Moreover, from a pragmatic perspective, we were concerned that individuals assigned to a waiting list condition would have little reason to remain involved and would therefore be more likely to be lost to follow-up. When using an intent-to-treat model that defines missing = smoking, such differential attrition would have biased results in favor of the intervention condition or type I error [53]. Thus, we used a control/comparison condition that offered a face-valid, credible, Web-based intervention for smoking cessation that did not contain what have been shown to be the active ingredients of evidence-based smoking cessation intervention. It is reasonable to question whether a better choice of control condition would have been a basic or static information website similar to what has been used in other RCTs of tobacco cessation interventions (eg, [16,19]).

Our remarkably successful use of online recruitment may have resulted in recruiting smokers who were less likely (1) to remain fully involved over time in a research program and (2) to quit smoking. It was quite easy for prospective participants to enroll—we estimate that it would require less than 15 minutes from clicking on a link in a Google ad listing to completing the screening, online consent, registration, and baseline assessment. The absence of measures describing ease of enrollment (either self-report or using a measure of convenience like elapsed time) makes it impossible for us to discuss further the extent to which the present study may have had easier enrollment than other Web-based behavioral interventions. Adding more barriers/hurdles to this process would very possibly have increased engagement and reduced program attrition because only motivated individuals would have been able to participate [54,55]. But, while culling out less motivated individuals, this approach might spuriously inflate absolute effect sizes while reducing external generalizability [56]. It remains for future research to determine the extent to which open online enrollment with few barriers for entry results in greater attrition.

Another likely limitation of the QSN intervention is that it may have been too expansive; that is, it may have offered too much (sometimes redundant) content that forced users to navigate through a complex information architecture, which reduced utility and encouraged attrition. We obtained ratings of program usability at follow-up, but, of course, interpretation of such data is constrained by the fact that they are drawn from a minority of original participants who completed follow-up assessments. Content duplication and/or complex intervention design can erode therapeutic impact—more is not necessarily better [57]. Indeed, the likely mechanisms of change may not be best described by a linear dose-response relationship [58,59].

In many ways, the results of the current research underscore the complexity of developing and then evaluating Web-based interventions for smoking cessation. We used an intervention and an attention placebo control that recommended physical activity as a smoking cessation tool. Our initial plan was to recruit through worksites as had been done with success in other Web-based smoking cessation research [18]. Because we found that worksite recruitment produced little in the way of results, we turned to online recruitment tied to popular Web search engines, particularly Google. Using this approach, we were able to rapidly randomize over 2300 participants to our RCT of two Web-based smoking cessation programs that had been carefully crafted to provide users with the content they needed in an online context that they would find interesting and engaging.

In summary, the results of this Web-based smoking cessation intervention RCT failed to confirm our hypotheses. Negative findings play an essential role in the development of science [60] and are particularly illuminating with regard to shaping a reasoned appreciation for the complexity of creating, delivering, and evaluating online health behavior change interventions. As we perform empirical tests of evolving interventions in this nascent field, we need to learn from both negative and positive outcomes as we strive to understand the factors that are associated with participant recruitment, program exposure, content tailoring, and participant attrition.

## Multimedia Appendix

PowerPoint presentation of program screenshots

## Abbreviations

PAR-Q: Physical Activity Readiness Questionnaire
QSN: Quit Smoking Network
RCT: randomized controlled trial
SHIP: Smokers’ Health Improvement Program
