# Supplementary material for: Comparing Two Web-Based Smoking Cessation Programs: Randomized Controlled Trial
Source: J Med Internet Res. 2008 Nov 18;10(5):e40. doi: 10.2196/jmir.993 (PMC2630830; doi:10.2196/jmir.993)
Supplement: Supplementary file 1 [file jmir_v10i5e40_app1.ppt]

## Slide 1
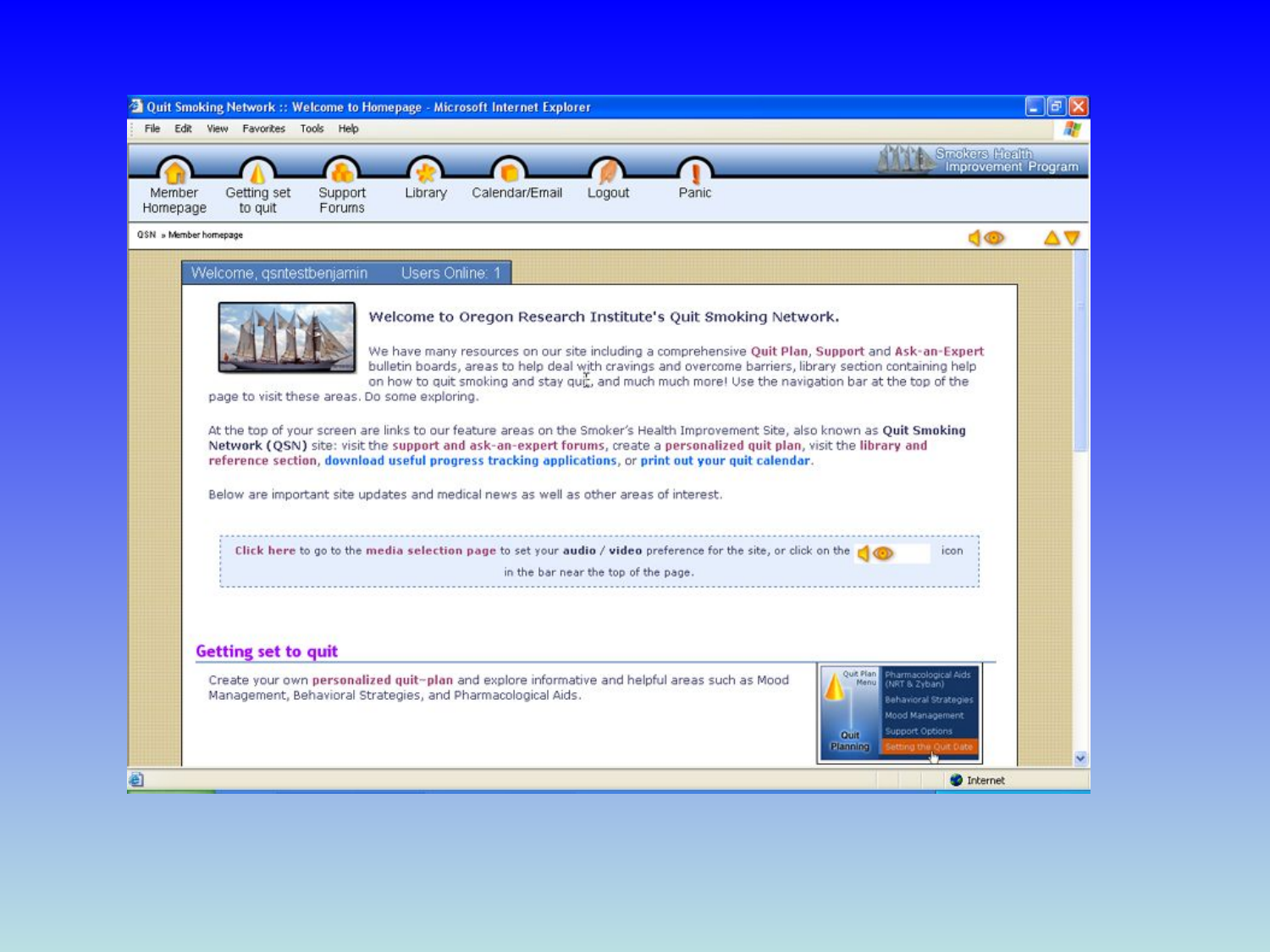

## Slide 2
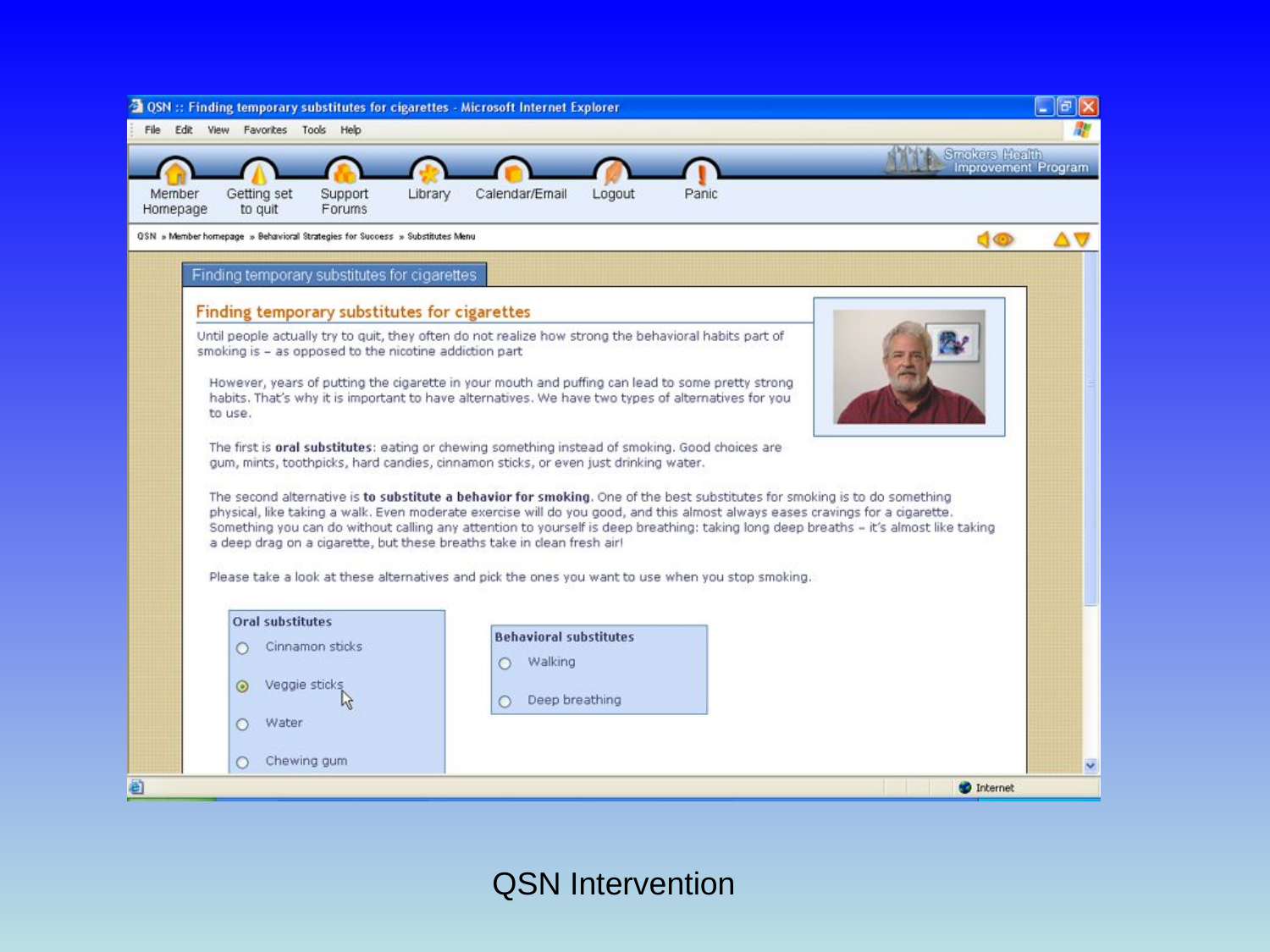

QSN Intervention

## Slide 3
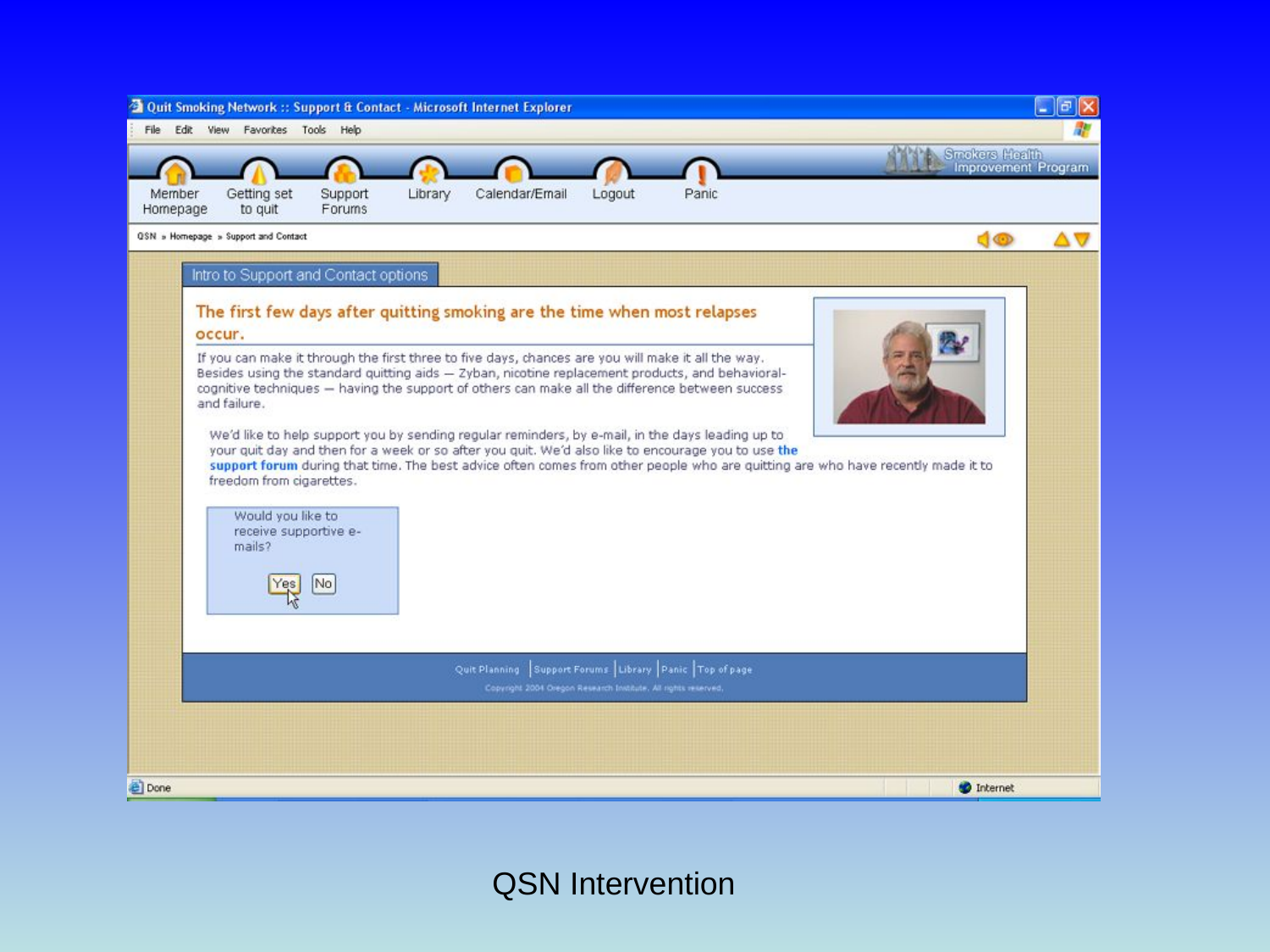

QSN Intervention

## Slide 4
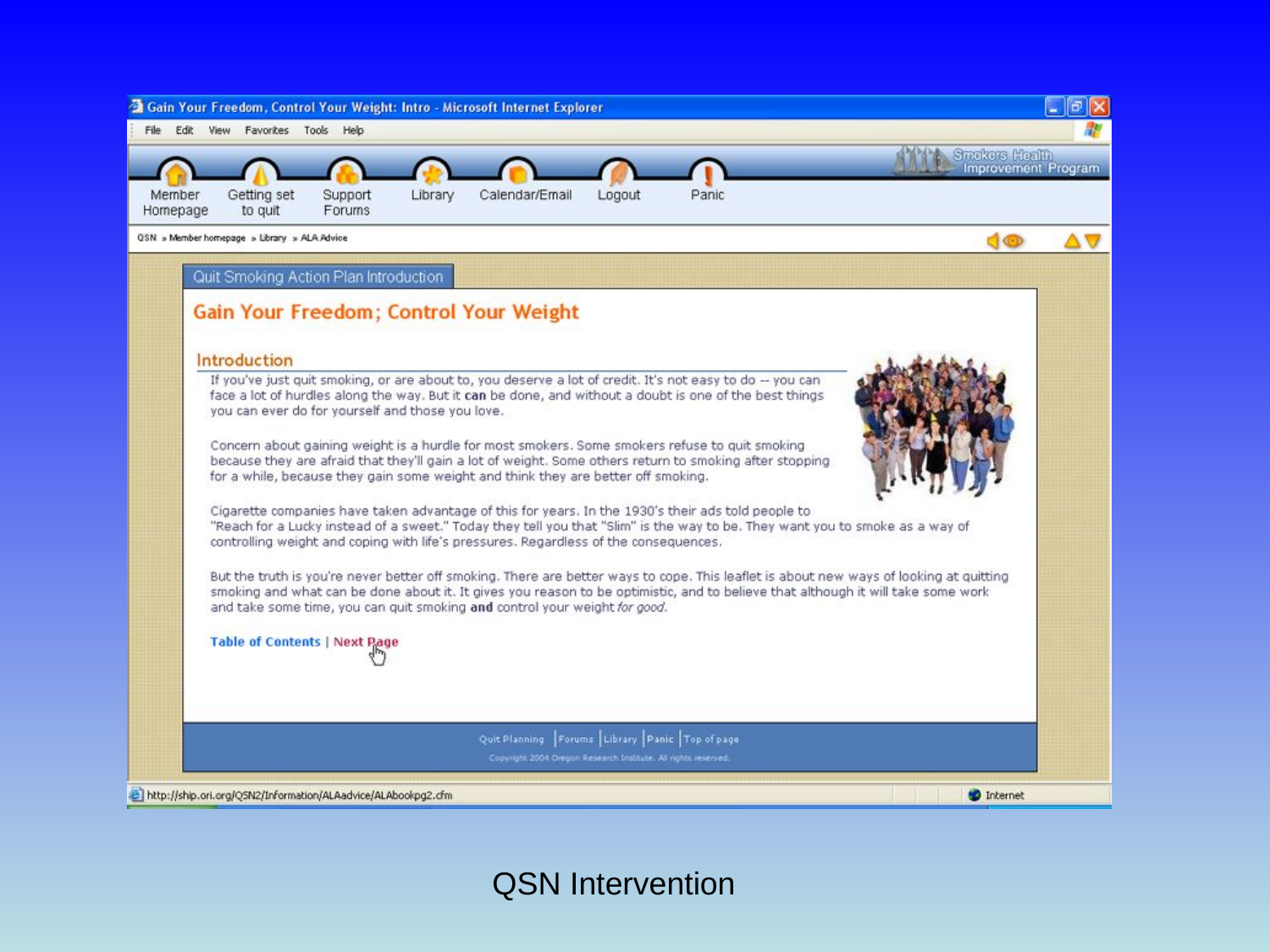

QSN Intervention

## Slide 5
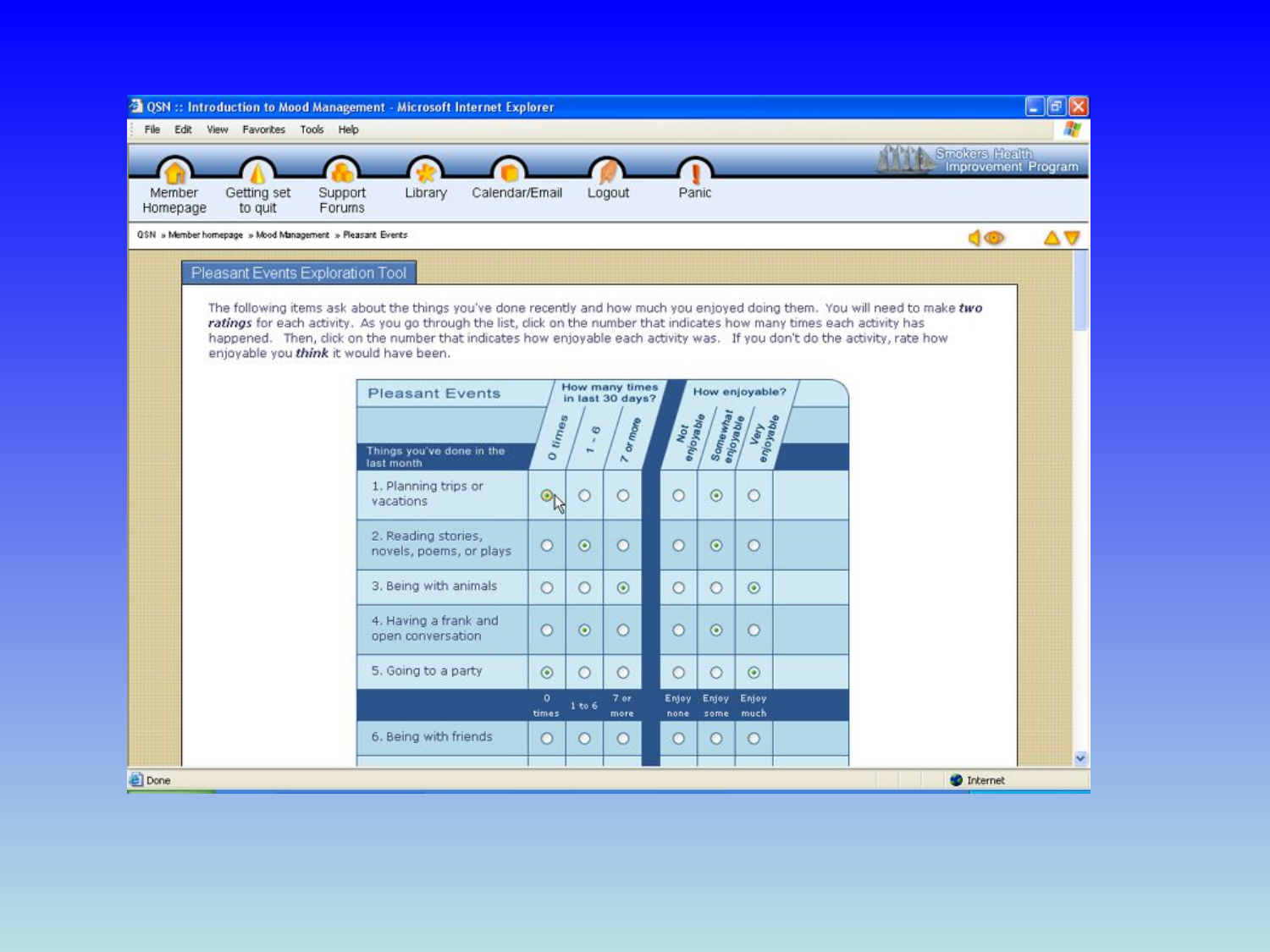

## Slide 6
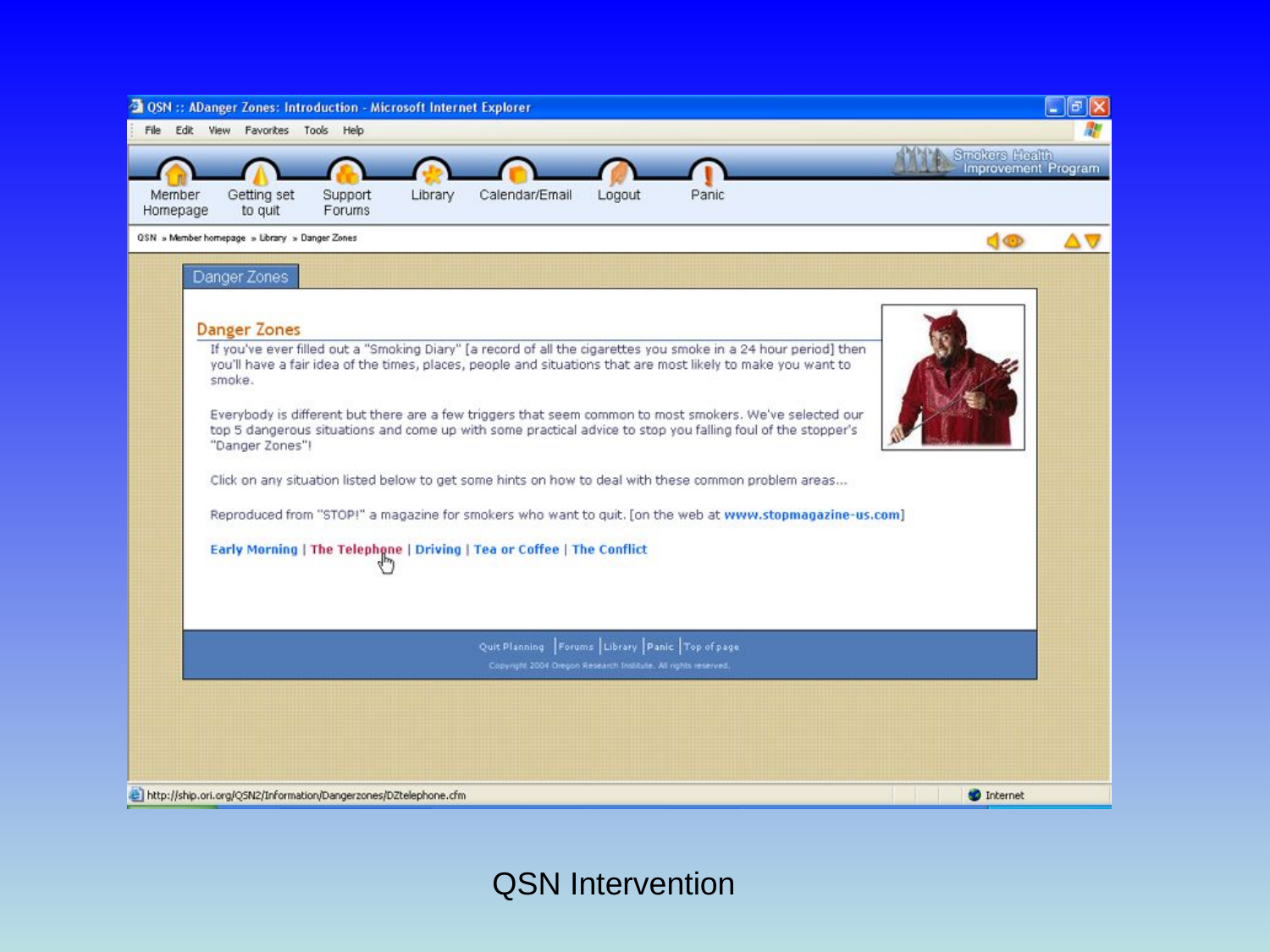

QSN Intervention

## Slide 7
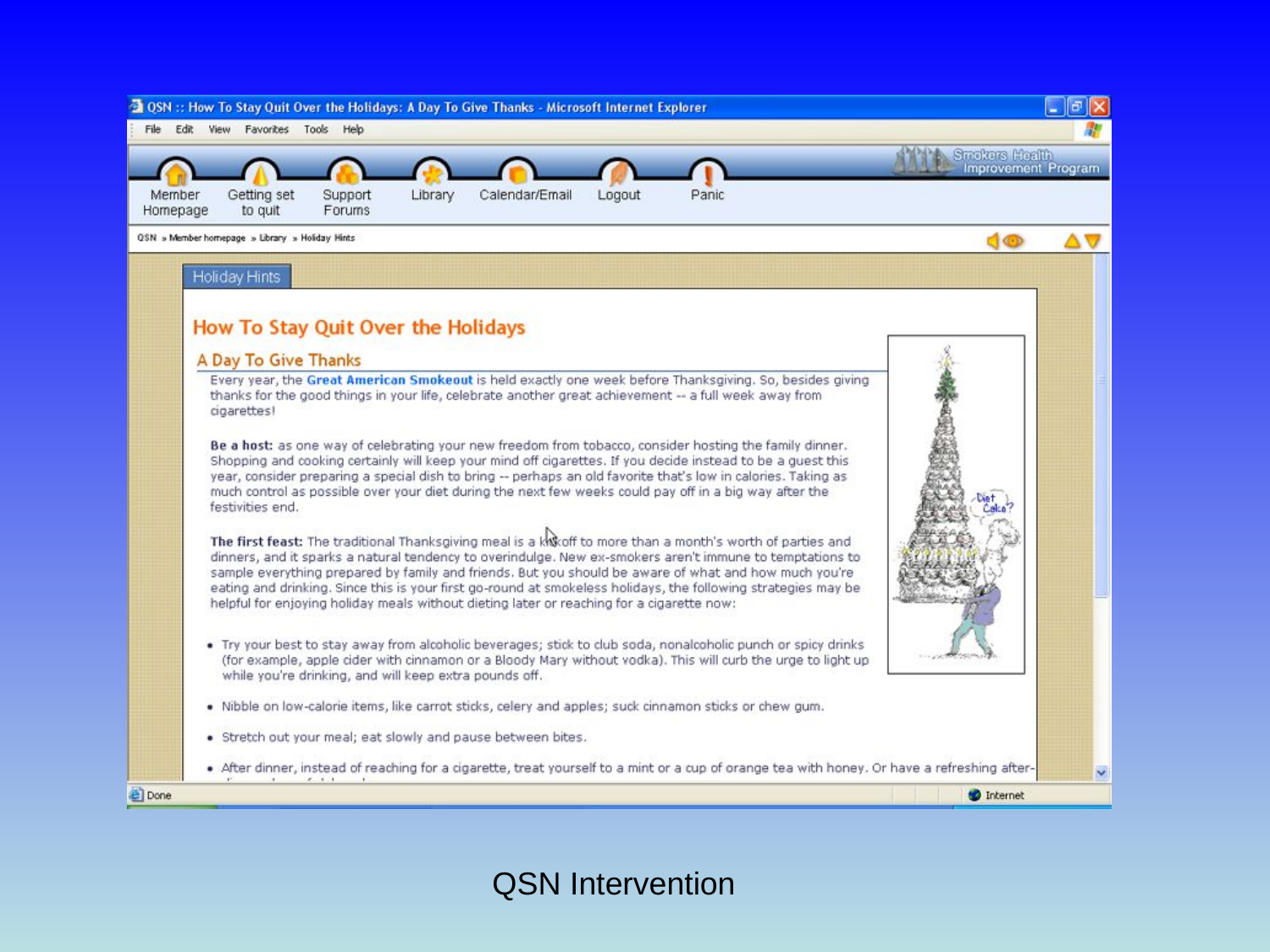

QSN Intervention

## Slide 8
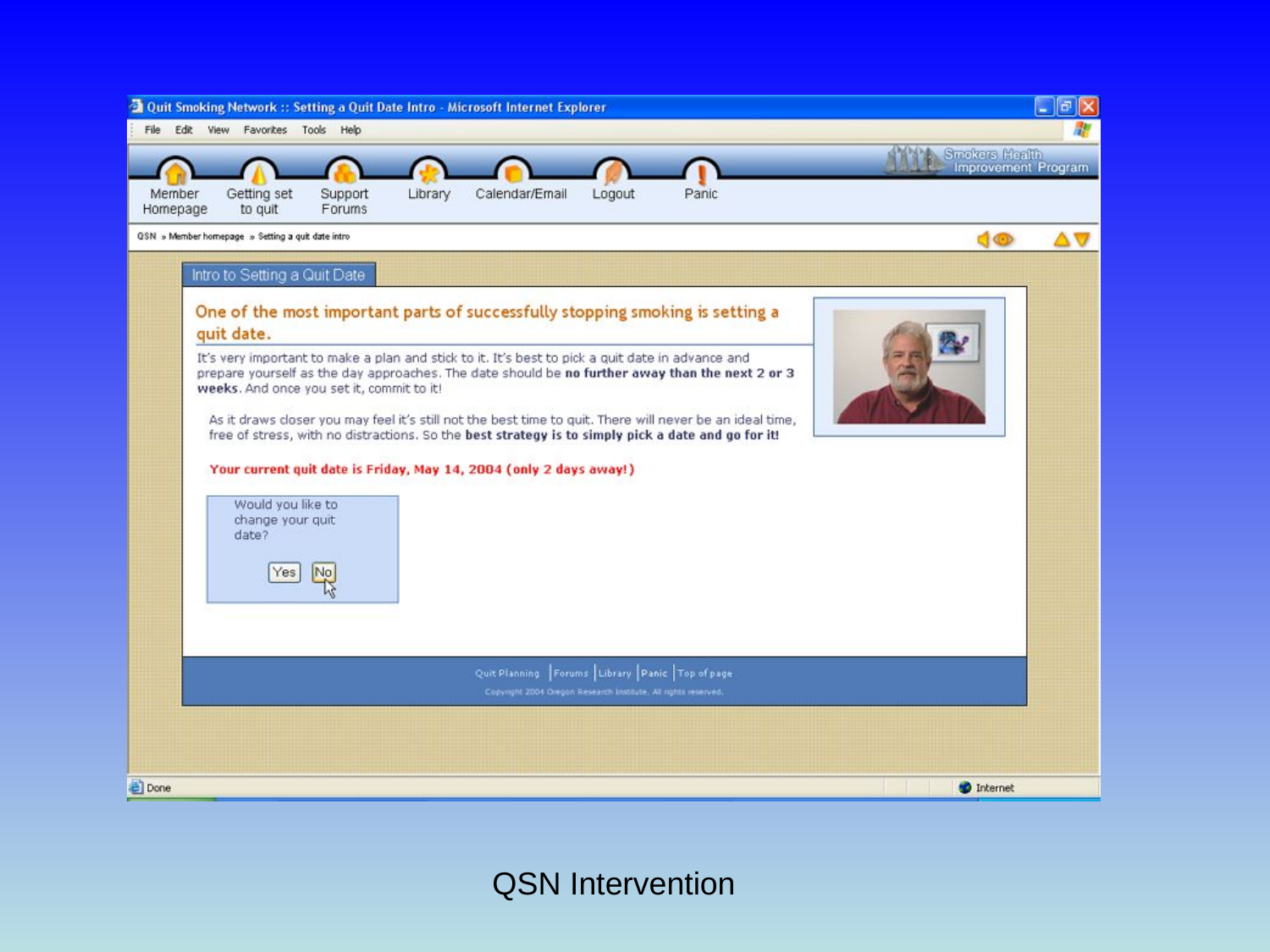

QSN Intervention

## Slide 9
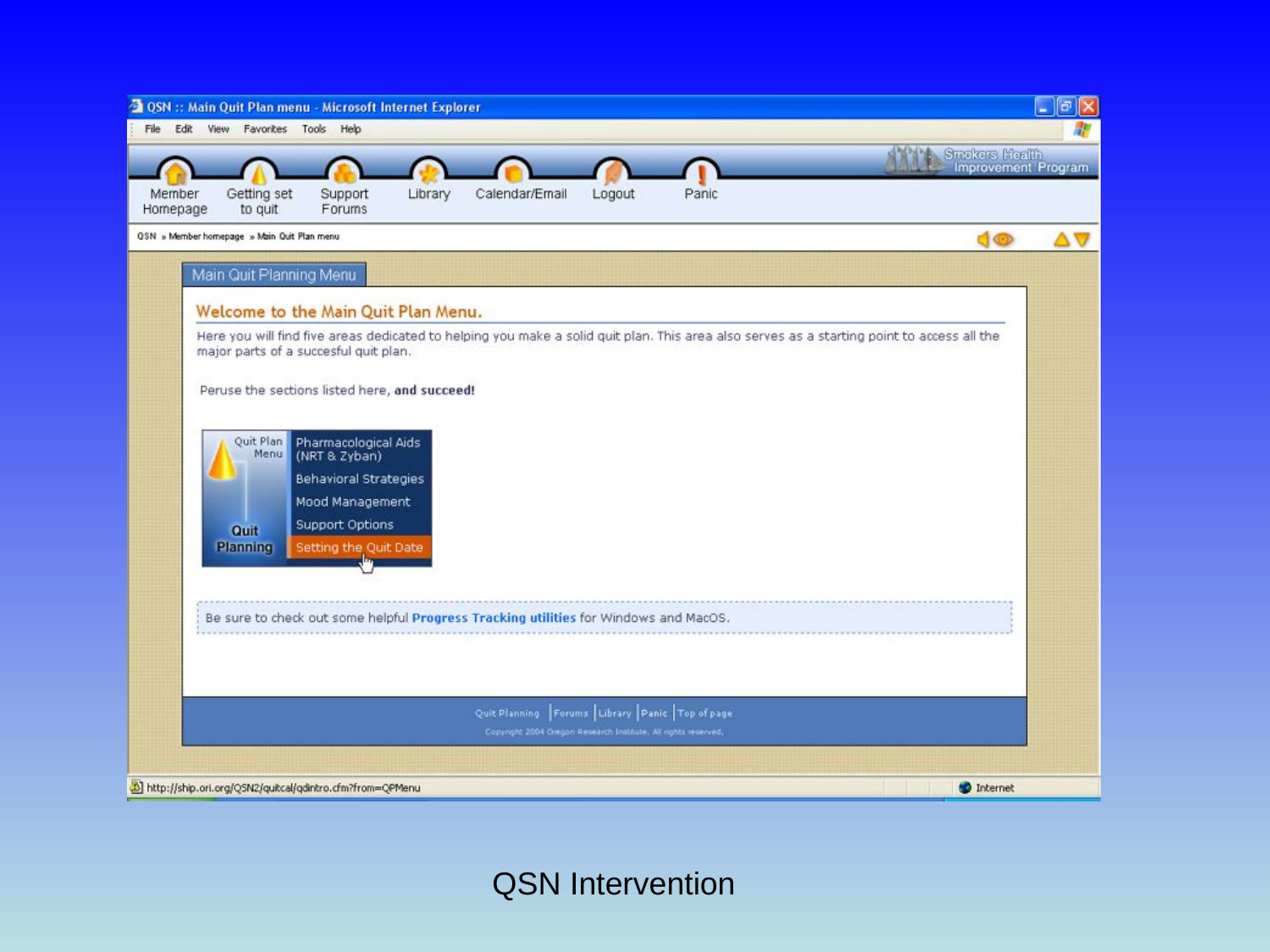

QSN Intervention

## Slide 10
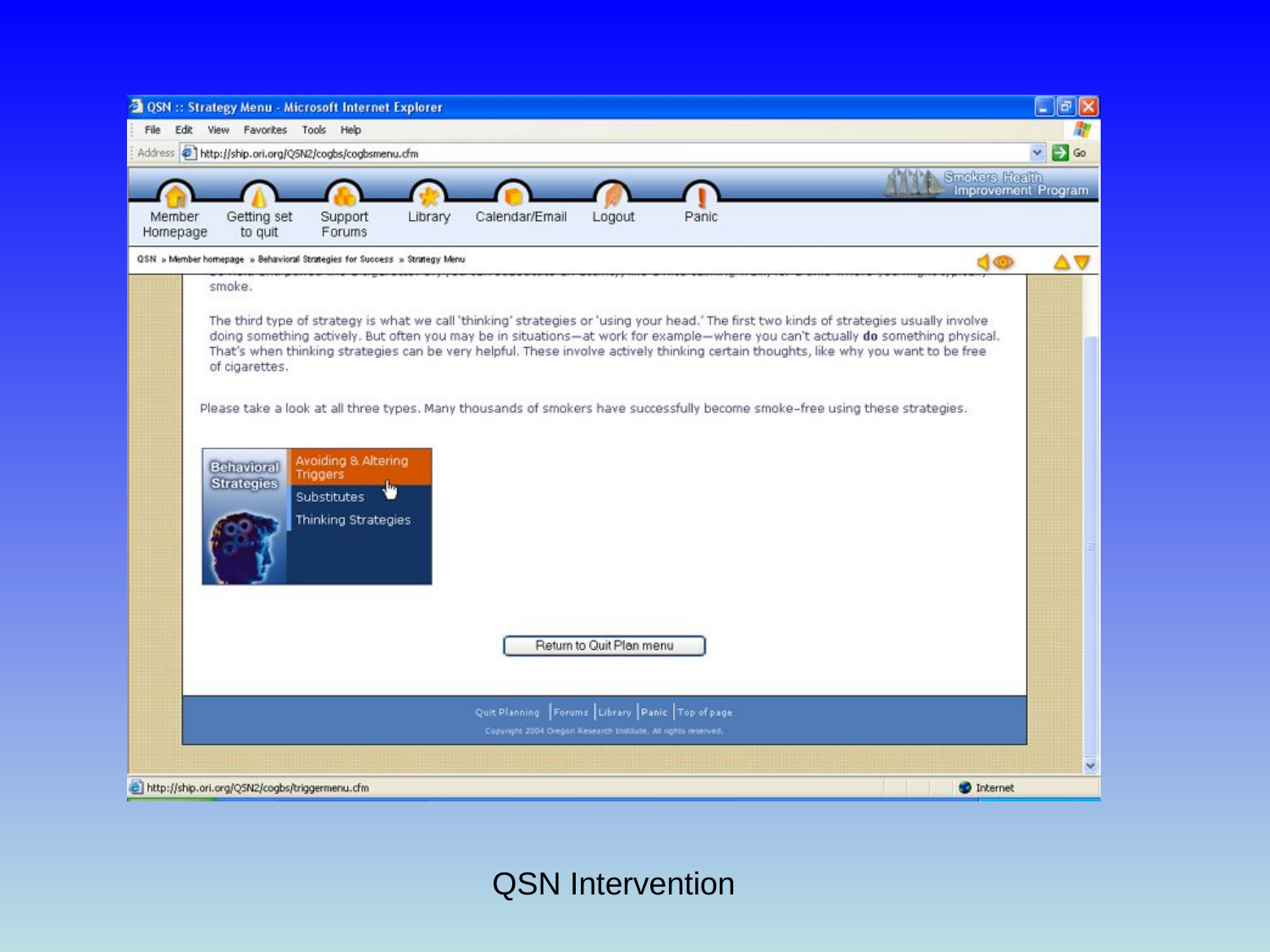

QSN Intervention

## Slide 11
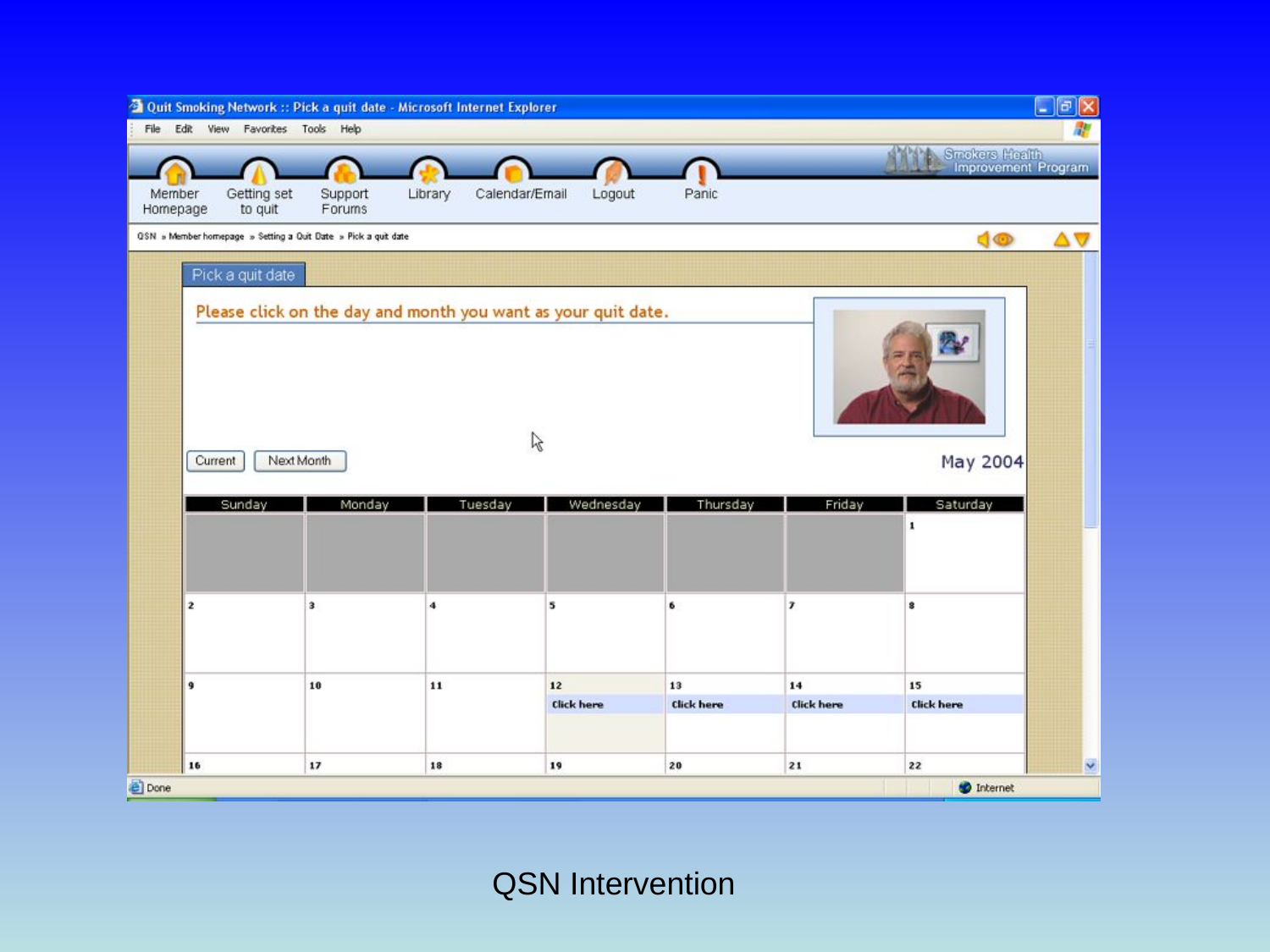

QSN Intervention

## Slide 12
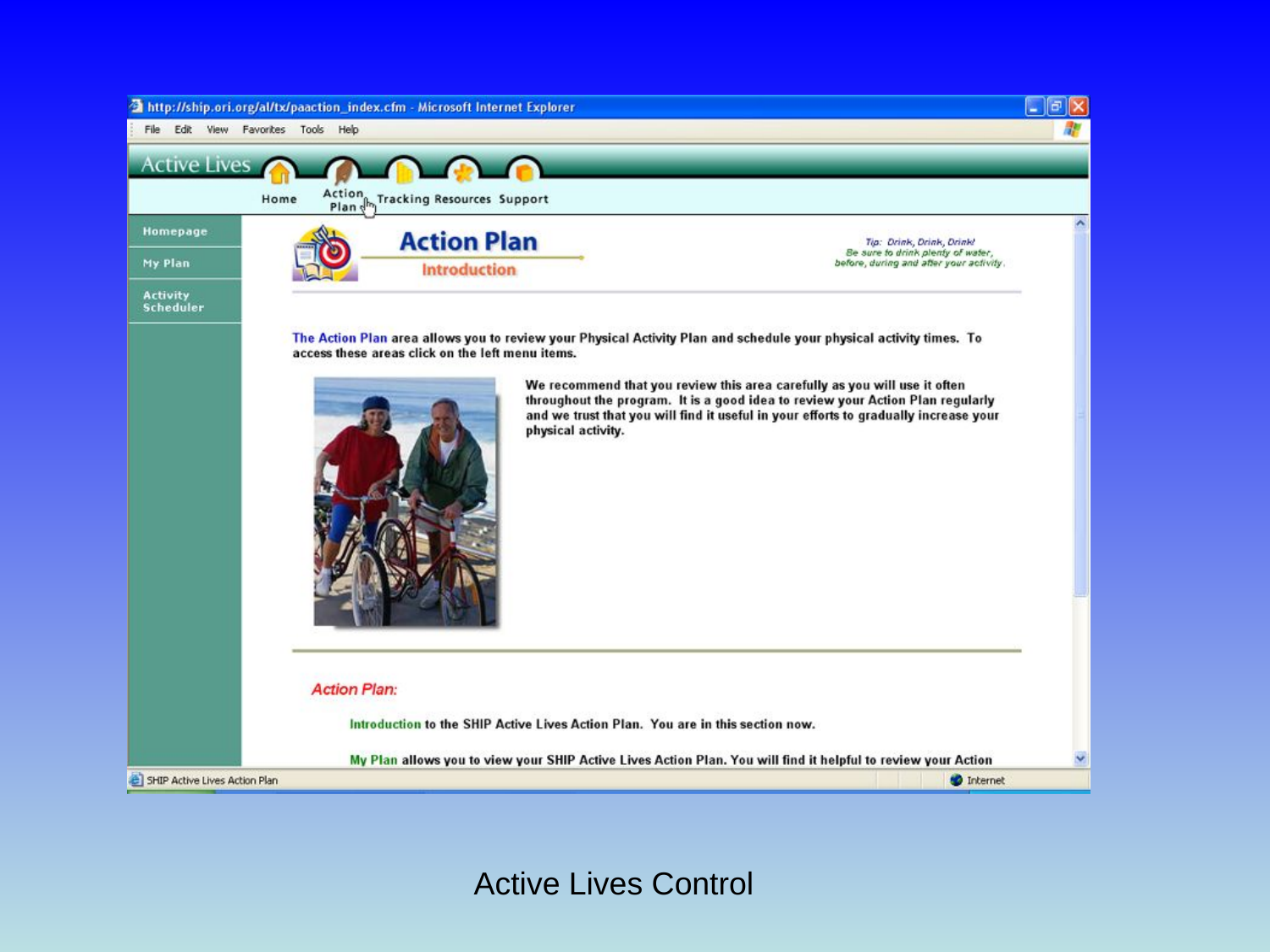

Active Lives Control

## Slide 13
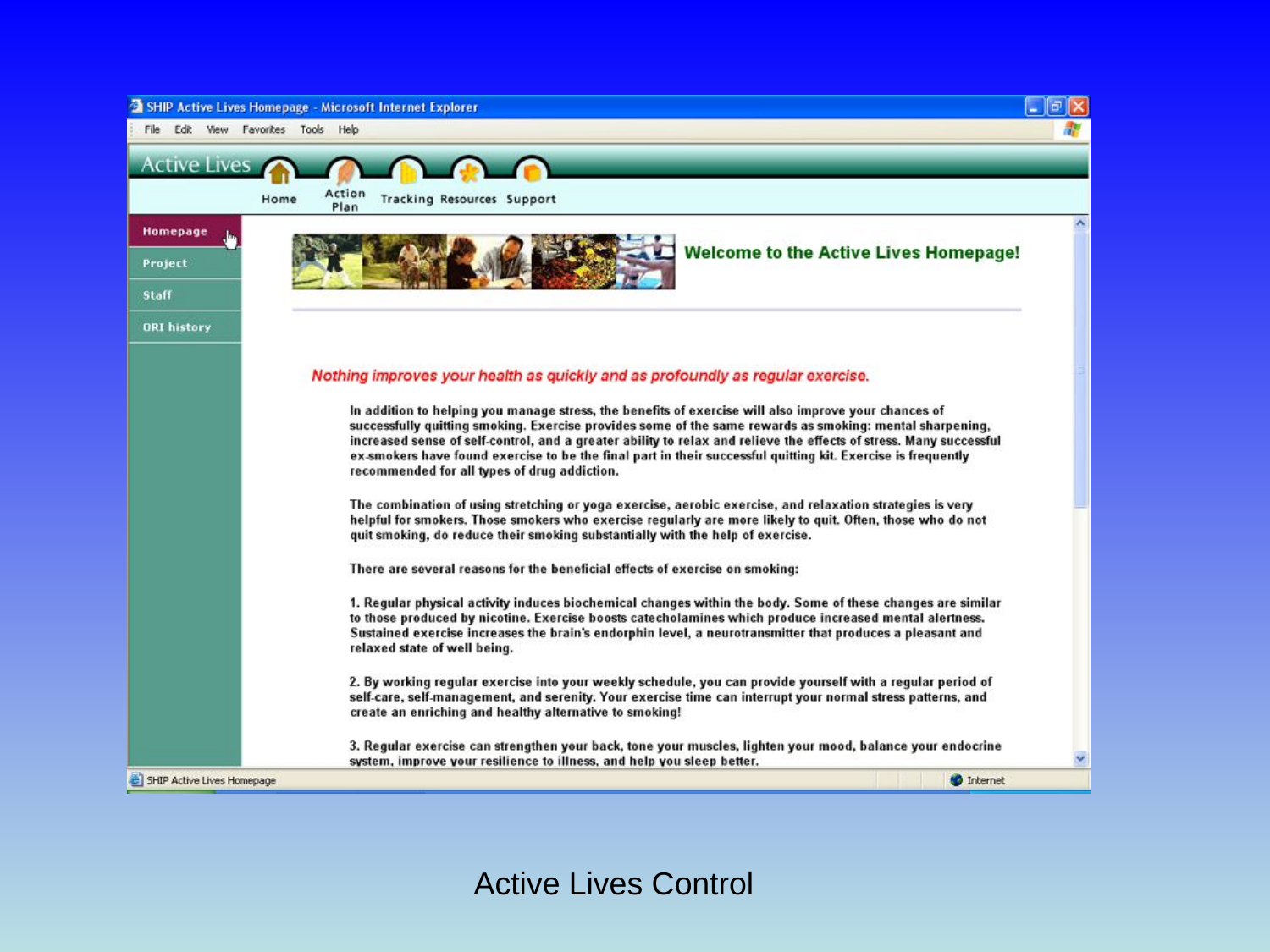

Active Lives Control

## Slide 14
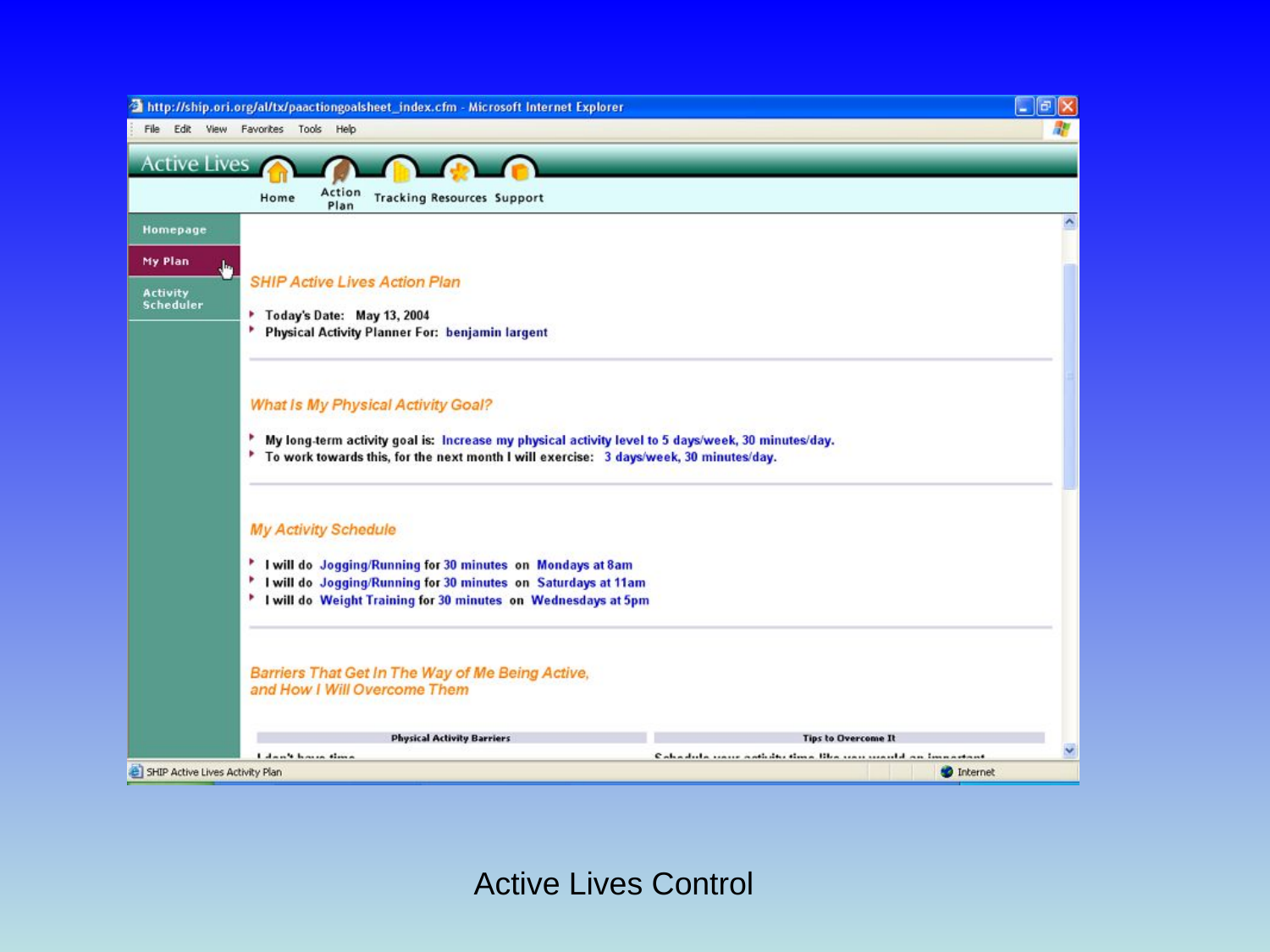

Active Lives Control

## Slide 15
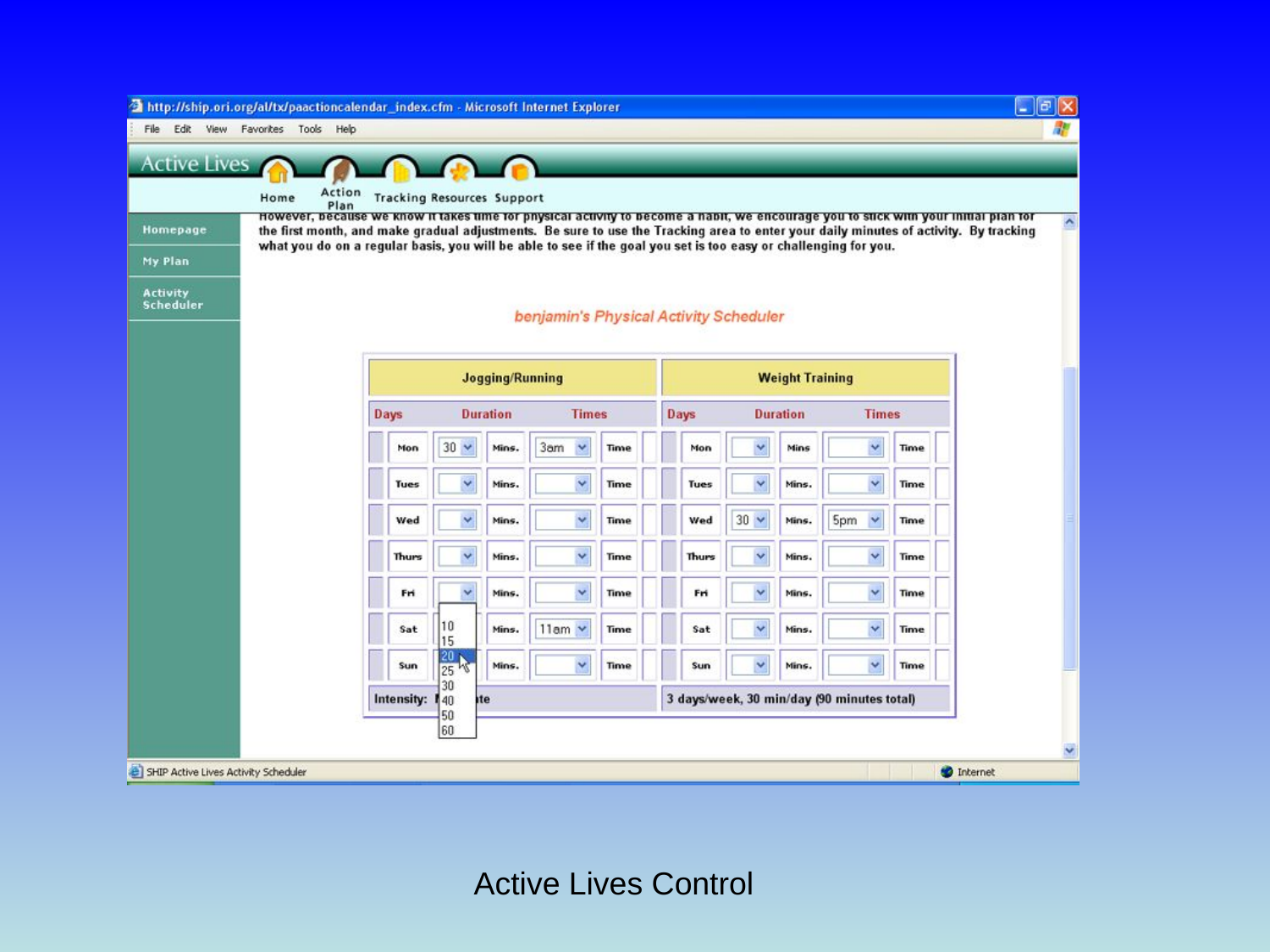

Active Lives Control

## Slide 16
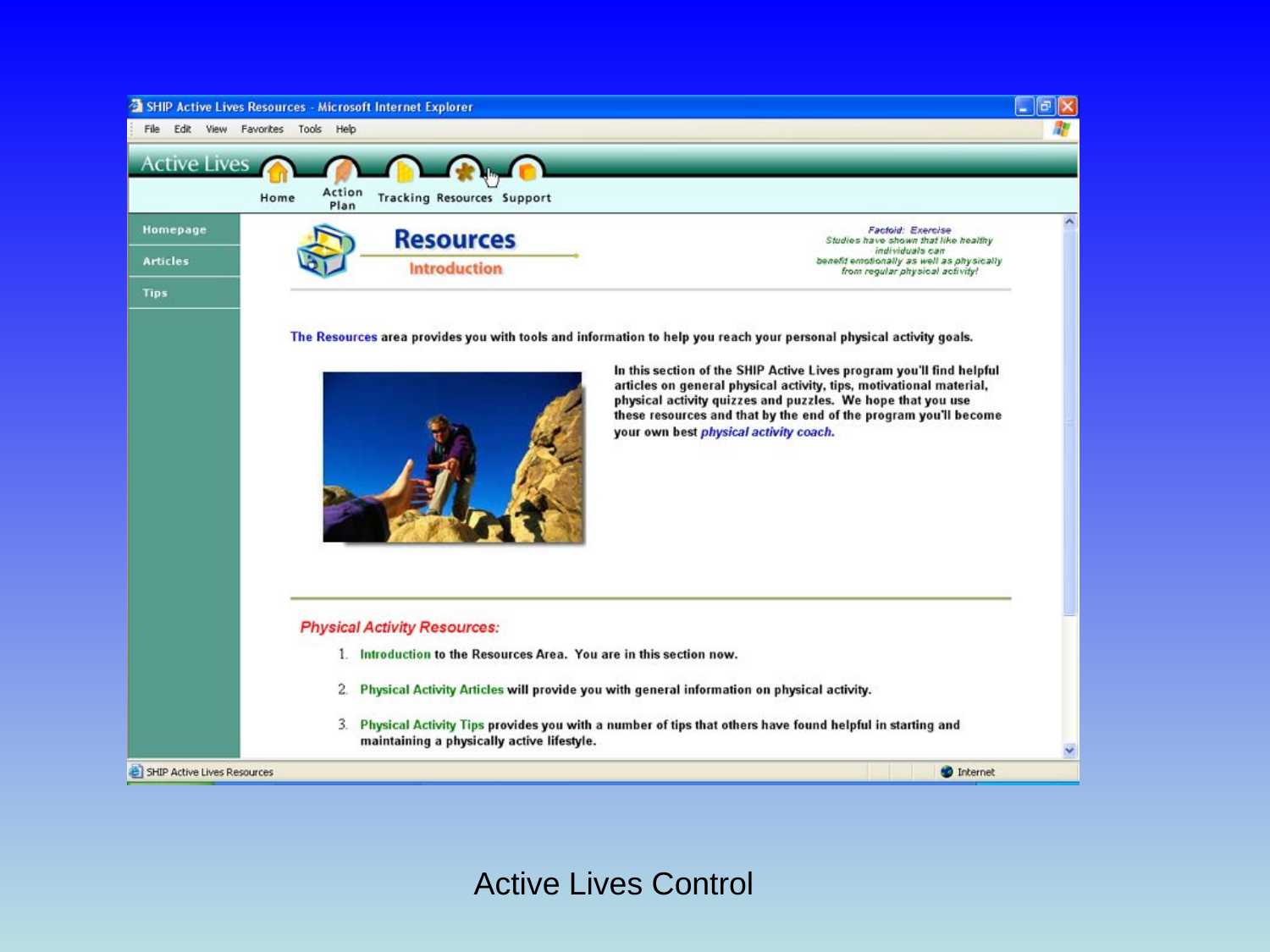

Active Lives Control

## Slide 17
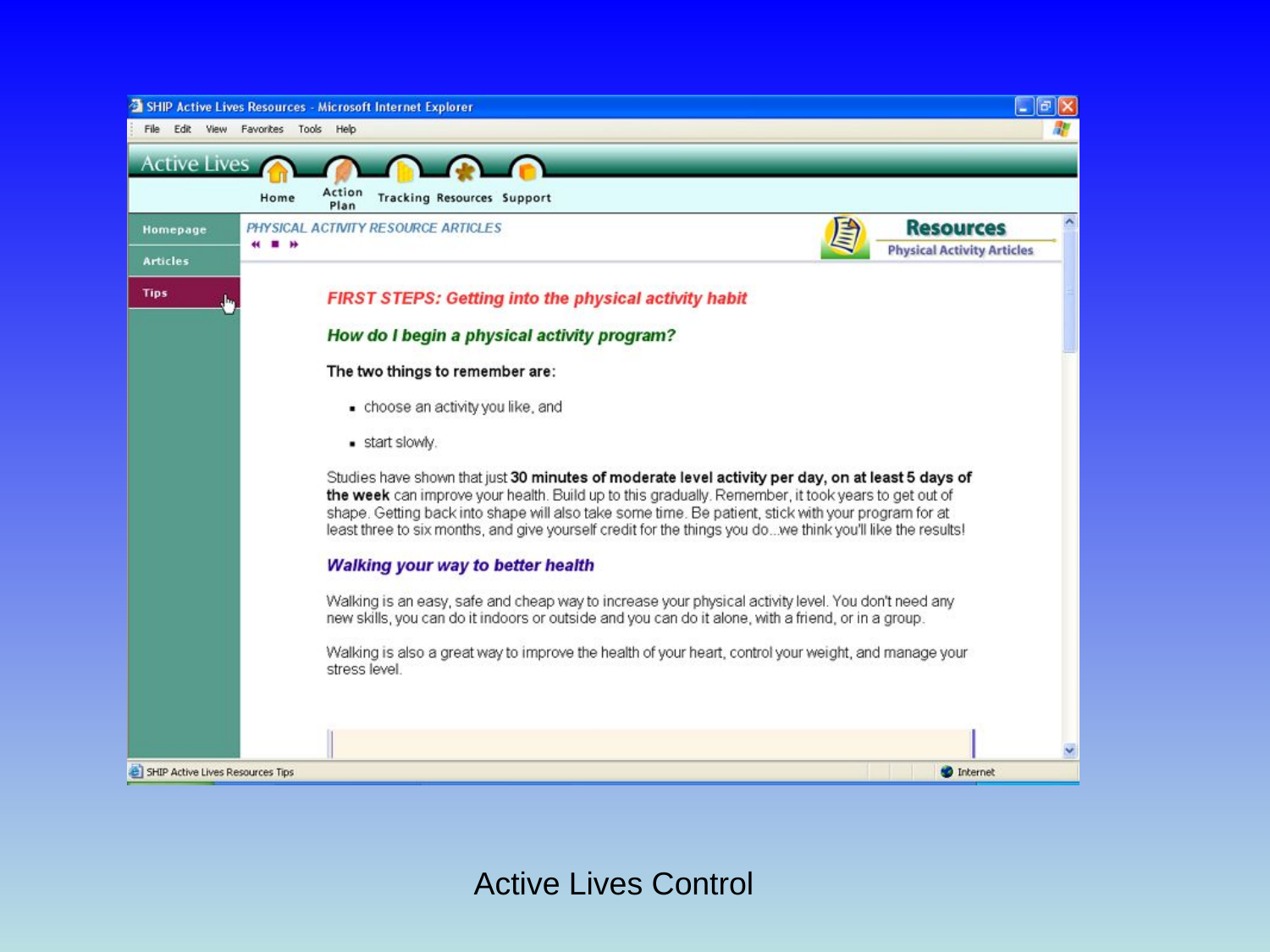

Active Lives Control

## Slide 18
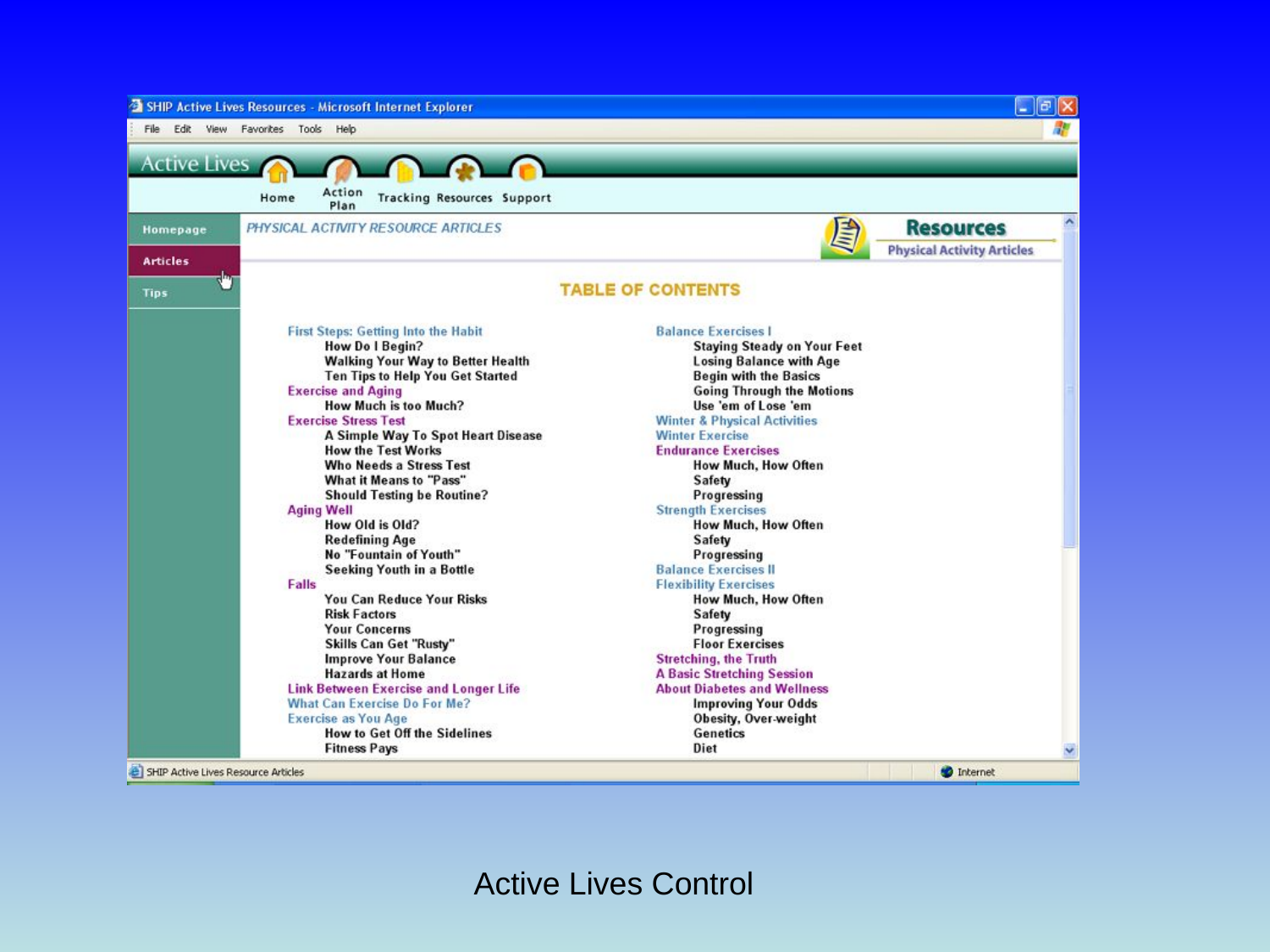

Active Lives Control
